# The Association of Early Childhood Cognitive Development and Behavioural Difficulties with Pre-Adolescent Problematic Eating Attitudes

**DOI:** 10.1371/journal.pone.0104132

**Published:** 2014-08-07

**Authors:** Rebecca C. Richmond, Oleg Skugarevsky, Seungmi Yang, Michael S. Kramer, Kaitlin H. Wade, Rita Patel, Natalia Bogdanovich, Konstantin Vilchuck, Natalia Sergeichick, George Davey Smith, Emily Oken, Richard M. Martin

**Affiliations:** 1 School of Social and Community Medicine, University of Bristol, Bristol, United Kingdom; 2 MRC Integrative Epidemiology Unit (IEU), University of Bristol, Bristol, United Kingdom; 3 Psychiatry and Medical Psychology Department, Belarussian State Medical University, Minsk, Belarus; 4 Departments of Pediatrics and of Epidemiology, Biostatistics and Occupational Health, McGill University Faculty of Medicine, Montreal, Canada; 5 The National Research and Applied Medicine Mother and Child Centre, Minsk, Belarus; 6 Obesity Prevention Program, Department of Population Medicine, Harvard Medical School and the Harvard Pilgrim Health Care Institute, Boston, Massachusetts, United States of America; 7 National Institute of Health Research (NIHR) Bristol Biomedical Research Unit in Nutrition, University Hospitals Bristol NHS Trust and University of Bristol, Bristol, United Kingdom; Harvard School of Public Health, United States of America

## Abstract

**Objectives:**

Few studies have prospectively investigated associations of child cognitive ability and behavioural difficulties with later eating attitudes. We investigated associations of intelligence quotient (IQ), academic performance and behavioural difficulties at 6.5 years with eating attitudes five years later.

**Methods:**

We conducted an observational cohort study nested within the Promotion of Breastfeeding Intervention Trial, Belarus. Of 17,046 infants enrolled at birth, 13,751 (80.7%) completed the Children's Eating Attitude Test (ChEAT) at 11.5 years, most with information on IQ (n = 12,667), academic performance (n = 9,954) and behavioural difficulties (n = 11,098) at 6.5 years. The main outcome was a ChEAT score ≥85^th^ percentile, indicative of problematic eating attitudes.

**Results:**

Boys with higher IQ at 6.5 years reported fewer problematic eating attitudes, as assessed by ChEAT scores ≥85^th^ percentile, at 11.5 years (OR per SD increase in full-scale IQ = 0.87; 0.79, 0.94). No such association was observed in girls (1.01; 0.93, 1.10) (p for sex-interaction = 0.016). In both boys and girls, teacher-assessed academic performance in non-verbal subjects was inversely associated with high ChEAT scores five years later (OR per unit increase in mathematics ability = 0.88; 0.82, 0.94; and OR per unit increase in ability for other non-verbal subjects = 0.86; 0.79, 0.94). Behavioural difficulties were positively associated with high ChEAT scores five years later (OR per SD increase in teacher-assessed rating = 1.13; 1.07, 1.19).

**Conclusion:**

Lower IQ, worse non-verbal academic performance and behavioural problems at early school age are positively associated with risk of problematic eating attitudes in early adolescence.

## Introduction

Problematic eating attitudes are common in childhood and early adolescence, with prevalence estimates of up to 20% of girls aged 12–14 years[1] and similar estimates in boys.[2] Studies indicate that the prevalence of abnormal eating attitudes in non-Western countries is lower than that of Western countries but appears to be gradually increasing.[3] These unhealthy eating and weight-related attitudes do not meet the criteria for an eating disorder, but may have health-related consequences. In addition, adolescents showing problematic eating behaviours are predisposed to eating disorders later in life.[4]–[7] In one study, children who had early eating conflicts and struggles with food were at a six-fold increased risk of anorexia nervosa in later adolescence or young adulthood.[6] A number of social, familial, psychological, biological and genetic risk factors have been implicated in the aetiology of problematic eating,[7]–[10] including cognitive and behavioural problems.[11], [12]

The relationship between cognitive/behavioural difficulties and diagnosed eating disorders has been thoroughly investigated, where cognitive parameters measured using an array of clinical tests have been compared between controls and those with eating disorders.[13]–[15] However, few studies have evaluated the association of academic ability and psychosocial functioning with subclinical problematic eating attitudes in a general healthy population. Of those that have, these have generally been cross-sectional in nature [11], [16]–[18] so the directionality of associations between cognition and problematic eating attitudes cannot be accurately assessed. For example, poor nutrition could impair cognitive function[19], [20] while weight concerns could lead to psychological distress.[21] Therefore, rather than being risk factors, poor cognitive and psychosocial function may be a consequence of problematic eating (reverse causality).

Using data from a prospective follow-up study of 17,046 children recruited at birth in the Republic of Belarus, we aimed to investigate associations of cognitive ability and behaviour at age 6.5 years (identified using a validated IQ test, teacher-assessed academic performance and both parent- and teacher-assessed validated behavioural measures) with eating attitudes 5 years later.

## Materials and Methods

The study includes the children and mothers recruited to the Promotion of Breastfeeding Intervention Trial (PROBIT), who continued to attend interviews and examinations throughout the child's first year of life, and when they were aged 6.5 and 11.5 years of age. The original trial methods have been previously described.[22] In brief, PROBIT was a multi-centre, cluster-randomized controlled trial conducted in the Republic of Belarus. The experimental intervention was the promotion of increased breastfeeding duration and exclusivity, modeled on the Baby-Friendly Hospital Initiative.[23] Between June 1996 and December 1997, 17,046 mother-infant pairs were recruited during their postpartum hospital stay from 31 maternity hospitals and affiliated polyclinics, which had been randomly assigned to the promotion of breastfeeding experimental (n = 16) or control (n = 15) arm. Inclusion criteria specified that infants were full-term (≥37 weeks gestation), healthy, singletons who had a birth weight of at least 2.5 kg, with an Apgar score of ≥5 at 5 minutes postpartum, and that mothers were healthy and had initiated breastfeeding. The mother-infant pairs were followed up at scheduled intervals during infancy to 12 months of age (PROBIT I, n = 16,492 for a 96.7% response rate), at age 6.5 years (PROBIT II, n = 13,889 for an 81.5% response rate) and at 11.5 years (PROBIT III, n = 13,879 for an 81.4% response rate).

### Trial registration

Current Controlled Trials (www.controlled-trials.com/): ISRCTN37687716;

Clinicaltrials.gov (www.clinicaltrials.gov): NCT01561612

### Ethics Statement

PROBIT III was approved by the Belarussian Ministry of Health and received ethical approval from the McGill University Health Centre Research Ethics Board; the Human Subjects Committee at Harvard Pilgrim Health Care; and the Avon Longitudinal Study of Parents and Children (ALSPAC) Law and Ethics Committee. A parent or legal guardian provided written informed consent in Russian at enrolment and at the follow-up visit, and children provided written assent at the 11.5-year follow-up visit.

### Measurement of exposures

Interviews and examinations at 6.5 years were performed between 2002 and 2005 by one polyclinic pediatrician in each of 24 of the 31 polyclinics; in the remaining seven high-volume clinics, follow-up visits were shared by two pediatricians. One of the components of these visits was the administration of the Wechsler Abbreviated Scales of Intelligence (WASI).[24], [25] The WASI consists of four subtests of vocabulary, similarities, block designs and matrices. In our analysis, we grouped these subtests into verbal IQ (vocabulary and similarities), performance IQ (matrices and block designs) and full-scale IQ (all four subtests).

As previously reported, the WASI was translated from English to Russian and then back-translated to ensure comparability of the Russian version.[26] High inter-pediatrician agreement was achieved, with Pearson correlation coefficients (95% confidence intervals [CIs]) of 0.80 (0.67, 0.89) for vocabulary, 0.72 (0.54, 0.83) for similarities, 0.80 (0.67, 0.89) for block designs, and 0.79 (0.66, 0.88) for matrices, in a sample of 45 children). An audit was undertaken of 190 children in the study, who were retested at an average of 17.7 months after the initial polyclinic visit by a blinded psychologist or psychiatrist; the Pearson correlation coefficient for full-scale IQ was 0.70 (0.62, 0.76) when comparing the test results at the initial clinic visit with those at audit.[26] Children who had begun school by the time of their 6.5-year follow-up visit were also evaluated by their teachers in four academic subject areas: reading, writing, mathematics, and all other subjects. Based on items in the Teacher Report Form of the Child Behaviour Checklist,[27] each child was rated on a five-point Likert scale as far below (1), somewhat below, at, somewhat above, or far above (5) his or her grade level.

The Strengths and Difficulties Questionnaire (SDQ) was completed by both the parent (usually the mother) and teacher (for children who had started formal schooling) to assess the children's behaviour at age 6.5 years.[28] The SDQ is a brief scale devised for behavioural screening of children aged three to sixteen years [29] and contains 25 items, divided into five scales for conduct problems, hyperactivity/inattention, emotional symptoms, peer problems and pro-social behavior. Scores on the first four scales are combined to generate a total difficulties score ranging from 0 to 40. The teacher version of the SDQ is identical to that of the parent version. As previously reported, internal consistency of the teacher and parent responses was high (Cronbach's alpha 0.82 and 0.73 for total difficulties in the teacher and the parent SDQ, respectively), as was test–retest reproducibility of the parent SDQ (intra-class correlation coefficient 0.80 (95% CI: 0.74,0.85) for total difficulties), based on a random audit of 190 children. Spearman's correlations between the parent and teacher SDQ scores were modest (0.28 for total difficulties).[28]

### Measurement of eating attitudes

When the children were a median age of 11.5 years, they self-completed the Children's Eating Attitudes Test (ChEAT), a quantitative indicator of a number of problematic eating attitudes about weight, body image, food preoccupation, peer- and media- pressure, dieting, purging and control of food intake, all of which are related to eating disorders.[30] The ChEAT was completed during attendance of a research clinic follow-up and the children were not helped by their parents or the pediatrician. It was originally proposed as a 26-item questionnaire to assess these eating attitudes and behaviours on a Likert scale, ranging from 1 (always) to 6 (never). Owing to time constraints in the research clinic, and to simplify the questionnaire for the children, we modified the response options to a 3-item scale, and coded the respective responses as 3 (“often”), 1.5 (“sometimes”) and 0 (“never”). In addition, we excluded two of the original 26 questions from our analysis because they were inversely correlated with the total variance in ChEAT scores (question 19, “I can show self-control around food” and question 25, “I enjoy trying new rich foods”). One of these questions was similarly dropped in the study by Maloney et al for the same reason.[31] Our adapted ChEAT-24 gave a range of 0–72, a similar range of possible total scores to the original ChEAT-26 (0–78). We used principal components analysis to verify the factor structure of the ChEAT questionnaire, as described previously.[32]

The ChEAT-24 scores were positively skewed and had a bimodal distribution because 10% of children had a total score of 0 (i.e. answered “never” to all 24 questions). Therefore, as in previous studies[1], [30], [33]–[37] we could not enter ChEAT-24 scores as a continuous outcome in our regression models. Previous studies have used ChEAT scores ranging between the 75^th^ and 91^st^ percentiles in their data as thresholds suggestive of problematic eating attitudes.[1], [30], [33]–[37] We defined our primary outcome as a ChEAT-24 score ≥ the 85^th^ percentile in our data (≥22.5), because lower thresholds generate more false positives, especially in younger children.[38], [39] In a sensitivity analysis, we investigated associations using a ChEAT-24≥ the 91^st^ percentile (≥25.5).

In one polyclinic, an intervention site, 76% of the 928 respondents answered “never” to all 24 items of the ChEAT questionnaire and just 2.3% of individuals scored ≥22.5, the threshold for risk.[32] In a sensitivity analysis, this site was excluded to determine its influence on results.

### Measurement of potential confounders

We considered the following as potential confounders: geographical variables i.e. the hospital/polyclinic location (urban or rural, and West or East of Belarus); child variables, i.e. child's age at the measurement of ChEAT, sex, gestational age, birth weight, 5 minute Apgar score and body mass index (BMI) at age 6.5 years; family variables, i.e. mother's age at the child's birth, parental education, highest household occupation, number of older children in household and maternal smoking during pregnancy. Treatment arm was also taken into account, and provided a measure of breastfeeding, since the intervention arm substantially increased duration and exclusivity of breastfeeding.[22] Inclusion of this variable as a potential confounder was also important as we have shown the randomized PROBIT treatment arm to be associated with both IQ at age 6.5 years [26] and ChEAT score at age 11.5 years.[40]

### Statistical Analysis

We estimated multilevel, mixed-effects logistic regression models using the ‘xtmelogit’ command in STATA version 12 (STATA Corp, Texas), to appropriately account for potential non-independence and clustering of measurements collected within an individual hospital/polyclinic site. In addition, for teacher-assessed measures, the models also accounted for clustering of teachers within the hospitals and polyclinics.

We investigated associations of our main exposures of interest (IQ, academic performance and behavioural difficulties) with ChEAT score ≥85^th^ percentile (≥91^st^ percentile in sensitivity analyses). The following multivariable mixed-effects logistic regression models were built: a basic model controlling for both age and sex, accounting for clustering by hospital/polyclinic and, as appropriate, for teacher; and an adjusted model, controlling for all potential confounding factors found to be associated with the ChEAT score and accounting for clustering. The regression coefficients are ORs per standard deviation (SD) increase in IQ; per unit increase in teacher-assessed academic performance; and per SD increase in SDQ score (total and subscore).

*P*-values for heterogeneity were calculated for binary and unordered categorical exposures. *P*-values for trend were calculated for all continuous and ordered categorical exposures, entered as linear terms into the models. Likelihood ratio tests were used to assess the assumption of linearity in these models. The assumption of linearity was satisfied for all the main exposure variables.

We tested whether the associations of our exposures with problematic eating attitudes differed in boys versus girls using likelihood ratio tests for interaction in the logistic regression models. For most analyses we found little evidence of interaction, so we present associations among girls and boys combined. However, there was evidence for interaction between each of the IQ measures and sex on ChEAT scores (p≤0.04) and thus associations of full-scale and sub-test IQ with ChEAT scores were stratified by sex. All analyses were conducted using STATA version 12 (STATA Corp, Texas).

## Results

**Figure 1:**
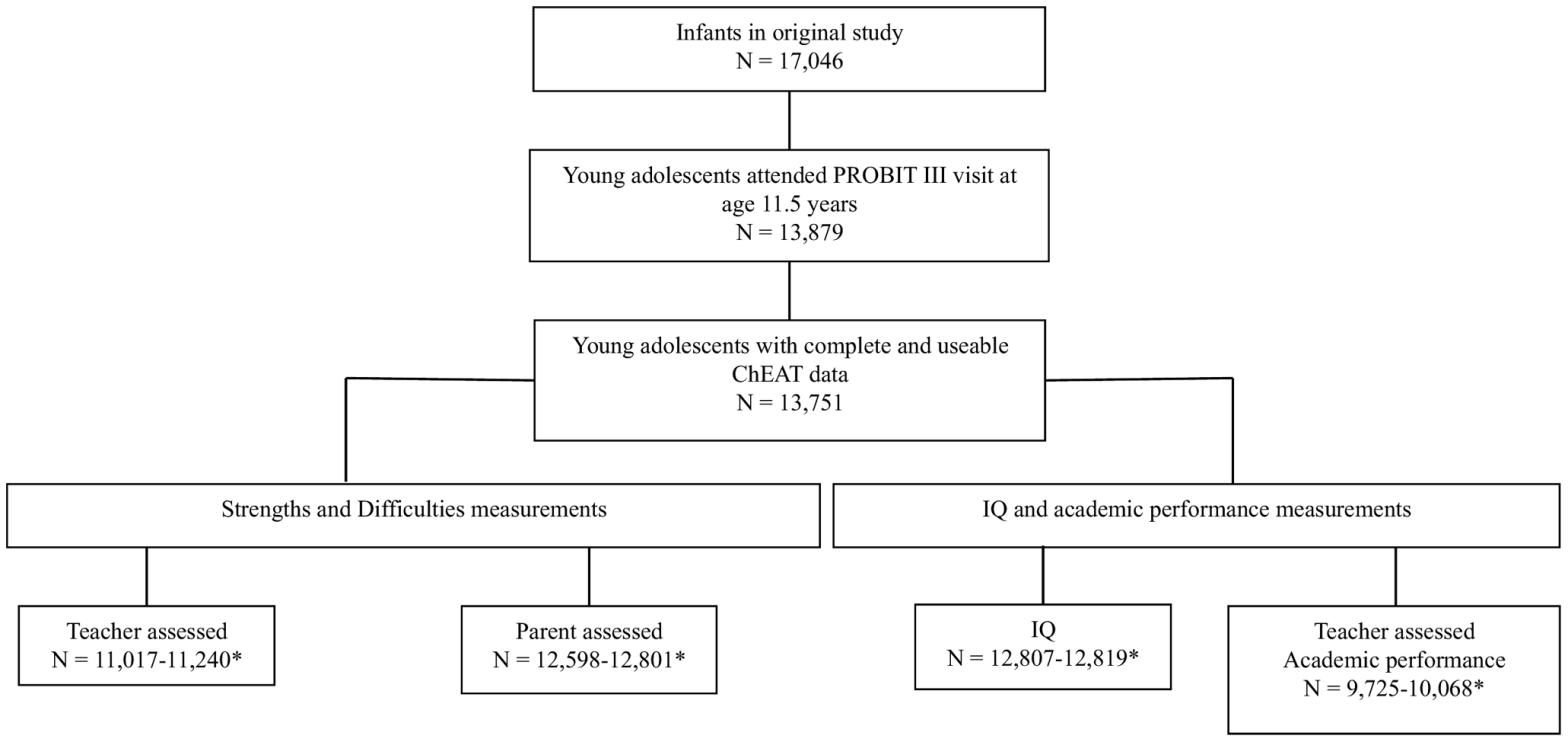
Flow chart of participant sample sizes for each exposure, PROBIT cohort. *Sample size varies depending on the completeness of data collection for each of the sub-areas of the cognitive/behavioural assessments. Final numbers included in the analyses were reduced slightly due to some missingness of the covariables.

Of the 17,046 children enrolled in the original trial, a total of 13,879 (81.4%) were seen at age 11.5 years (IQR 11.3–11.8) and 13,751 (80.7%) had complete and useable responses to the ChEAT questionnaire (6675 girls and 7076 boys). Complete cases analysis was used throughout and so the sample sizes on which our analyses are based vary depending on the completeness of data collection when the children were aged 6.5 years (**Figure 1**). A comparison of characteristics of the mother-infant pairs that were not followed-up and of those who were followed-up when the offspring were age 11.5 years has been previously published.[41] Mothers who did not attend the follow-up visit were slightly younger at the time of birth of their infant, were slightly less likely to have partly completed university or advanced secondary education, and were more likely to have smoked during pregnancy, and the study child was more likely to have been their first child.

**Table 1** shows the descriptive characteristics of the cohort. The mean full-scale IQ at age 6.5 years was 106.0, at the upper end of the “average” classification for the Wechsler intelligence tests.[25] The mean parent and teacher SDQ total difficulties scores for the children were 11.5 and 9.6 respectively, close to the previously reported average.[29] Girls were more likely than boys to have ChEAT scores ≥85^th^ percentile (20.8% versus 14.1%, p<0.001).

**Table 1:** Descriptive characteristics of the PROBIT Cohort.

Baseline characteristics	Percentages (%), mean (SD) or median (IQR) (N = 13,751)
Female (%)	48.5
Median (IQR) age at physical examination (years)	11.5 (11.3–11.8)
Urban vs. Rural (% in Urban)	57.9
West vs. East of Belarus (% in West)	52.6
PROBIT (% within intervention arm)	53.5
Mean (SD) birth weight (g)	3442.5 (420.1)
Mean (SD) gestational age (weeks)	39.4 (1.0)
Apgar score at 5 minutes (% less than 9)	41.96
Highest Household Occupation:	
Unemployed	5.4
Pupil/Student	1.3
Manual worker	40.5
Service worker	52.8
Mother's education (% completed university)	13.6
Father's education (% completed university)	13.2
Mean (SD) age of mother at birth (years)	25.0 (4.9)
Number of older children in household (% one or more)	43.1
Maternal smoking in pregnancy (% 1 or more cigarettes a day)	2.1
Mean (SD) child BMI (kg/m^2^) at age 6.5 years	15.5 (1.6)
Mean (SD) full-scale IQ at 6.5 years	106.0 (16.1)
Mean (SD) total difficulties score in Parent SDQ (0–40)	11.5 (5.0)
Mean (SD) total difficulties score in Teacher SDQ (0–40)	9.6 (5.8)
Mean (SD) ChEAT score (continuous)	13.3 (8.9)
ChEAT score ≥85^th^ percentile (% score 22.5)	17.3

ChEAT  =  Children's Eating Attitudes Test; PROBIT  =  Promotion of Breastfeeding Intervention Trial; SDQ  =  Strengths and difficulties questionnaire.

Male sex (OR  = 0.62; 95% CI: 0.56, 0.68), age of child (OR per tertile  = 0.90; 0.85, 0.96), intervention group (OR  = 0.47; 0.24, 0.95), BMI of the child at age 6.5 (OR per tertile  = 1.20; 1.17, 1.24) and number of older children in the household (OR per category  = 0.90; 0.83, 0.97) were associated with ChEAT score ≥85^th^ percentile (**Table S1**). The confounding structure was similar when associations of potential confounders with ChEAT scores were analysed separately in boys and girls (results not shown).

**Table 2** shows the basic and adjusted models for associations of IQ measured at 6.5 years with ChEAT scores ≥85^th^ percentile at 11.5 years. In the adjusted model for boys and girls combined, there was some evidence of an inverse association of full-scale IQ (OR  = 0.94; 0.89, 1.00), verbal IQ (OR  = 0.95; 0.89, 1.00) and performance IQ (OR  = 0.95; 0.90, 1.00) with ChEAT ≥85^th^ percentile. Stratified by sex, there was evidence of a reduction in odds of problematic eating attitudes for each SD increase in full-scale IQ (OR  = 0.87; 0.79, 0.94), verbal IQ (OR  = 0.88; 0.81, 0.96) and performance IQ (OR  = 0.88; 0.81, 0.95) in boys. For girls, the respective ORs were 1.01 (0.93, 1.10), 1.01 (0.93, 1.09) and 1.01 (0.94, 1.10) (p for sex- interaction  = 0.016, 0.036 and 0.005 for full, verbal and performance IQ respectively).

**Table 2:** Association between each IQ measure and ChEAT scores ≥85^th^ percentile.

IQ Measures	Percentage of ChEAT scores ≥22.5			
	Basic Model†			
Full IQ (n = 12,663)	Overall	Females	Males	P-value for sex*IQ interaction
Below average (n = 2,083, 941, 1,142*)	18.6	22.5	15.4	
Average (n = 6,019, 3,049, 2,970)	17.4	20.3	14.5	
Above average (n = 4,561, 2,176, 2,385)	17.5	21.8	13.6	
**Basic Model** † Odds ratio (95% CI) per SD increase; P-value for trend	0.97 (0.91, 1.02); 0.27	1.04 (0.96, 1.13); 0.33	0.89 (0.82, 0.97); 0.01	0.016
**Adjusted Model** *^‡^* Odds ratio (95% CI) per SD increase; P-value for trend	0.94 (0.89, 1.00); 0.045	1.01 (0.93, 1.10); 0.78	0.87 (0.79, 0.94); 0.002	0.016
**Verbal IQ (n = 12,667)**				
Below average (n = 2,783, 1,258, 1,525)	18.6	21.9	15.8	
Average (n = 5,752, 2,915, 2,837)	17.1	20.0	14.2	
Above average (n = 4,132, 1,994, 2,138)	17.7	22.3	13.5	
**Basic Model** † Odds ratio (95% CI) per SD increase; P-value for trend	0.98 (0.93, 1.04); 0.48	1.04 (0.96, 1.12); 0.37	0.92 (0.84, 1.00); 0.05	0.044
**Adjusted Model** *^‡^* Odds ratio (95% CI) per SD increase; P-value for trend	0.95 (0.89, 1.00); 0.07	1.01 (0.93, 1.09); 0.90	0.88 (0.81, 0.96); 0.009	0.036
**Performance IQ (n = 12,675)**				
Below average (n = 1,314, 583, 731)	19.6	25.6	14.9	
Average (n = 7,145, 3,617, 3,528)	17.5	20.0	14.9	
Above average (n = 4,216, 1,971, 2,245)	17.4	22.0	13.3	
**Basic Model** † Odds ratio (95% CI) per SD increase; P-value for trend	0.96 (0.91, 1.02); 0.18	1.03 (0.96,1.11); 0.43	0.89 (0.82,0.96); 0.006	0.004
**Adjusted Model** *^‡^* Odds ratio (95% CI) per SD increase; P-value for trend	0.95 (0.90, 1.00); 0.08	1.01 (0.94, 1.10); 0.69	0.88 (0.81, 0.95); 0.004	0.005

† ORs adjusted for age, sex and cluster (polyclinic site). *^‡^* ORs adjusted for age, sex, cluster (polyclinic site), treatment arm, child's BMI at age 6.5 years and number of older children in household * (n = x, y, z): x =  total number of children in group, y =  total number of females in group, z =  total number of males in group.

IQ measures have been categorized as “below average” (<90), “average” (90–109) and “above average”(>109), according to Weschler scale IQ classifications, for the presentation of results, although IQ was included as a continuous, standardized variable in mixed-effects logistic regression models.

Teacher-assessed academic performance in both mathematics and other subjects (unrelated to mathematics, reading or writing) were inversely associated with ChEAT scores ≥85^th^ percentile (**Table 3**). In the adjusted model in boys and girls combined, one unit increases in scores for mathematics and other subjects were associated with 12 % (OR  = 0.88; 0.82, 0.94) and 14 % (OR  = 0.86; 0.79, 0.94) reductions in odds of problematic eating attitudes, respectively. There was little evidence of associations of reading and writing ability with ChEAT ≥85^th^ percentile. The associations shown in **Table 3** were similar in boys and girls (p for sex-interaction ≥0.11).

**Table 3:** Association between Teacher Assessed Academic Performance and ChEAT scores ≥85^th^ percentile.

Academic subject	Percentage of ChEAT scores ≥22.5			
	Basic Model†			
Mathematics (n = 9,954)	Overall	Females	Males	P-value for sex*IQ interaction
Far below grade (n = 248, 98, 150*)	23.8	34.7	16.7	
Somewhat below (n = 1,055, 456, 599)	17.6	22.8	13.7	
At grade level (n = 5,369, 2,632, 2,737)	18.4	21.4	15.4	
Somewhat above (n = 2,873, 1,461, 1,412)	16.9	19.9	13.7	
Far above grade (n = 409, 193, 216)	15.4	18.7	12.5	
**Basic Model** † Odds ratio (95% CI) per SD increase; P-value for trend	0.89 (0.83–0.96); 0.003	0.84 (0.76, 0.93); 0.002	0.95 (0.86, 1.06); 0.36	0.11
**Adjusted Model** *^‡^* Odds ratio (95% CI) per SD increase; P-value for trend	0.88 (0.82, 0.94); 0.001	0.83 (0.75, 0.92); 0.001	0.93 (0.84, 1.04); 0.20	0.15
**Writing (n = 9,760)**				
Far below grade (n = 269, 88, 181*)	18.2	23.9	15.5	
Somewhat below (n = 1,009, 323, 686)	17.1	22.3	14.6	
At grade level (n = 5,707, 2,665, 3,042)	17.9	21.7	14.6	
Somewhat above (n = 2,433, 1,452, 981)	17.9	20.1	14.6	
Far above grade (n = 342, 220¸ 122)	16.7	20.0	10.7	
**Basic Model** † Odds ratio (95% CI) per SD increase; P-value for trend	0.94 (0.87, 1.01); 0.12	0.92 (0.83, 1.02); 0.12	0.98 (0.87, 1.09); 0.68	0.55
**Adjusted Model** *^‡^* Odds ratio (95% CI) per SD increase; P-value for trend	0.93 (0.86, 1.00); 0.06	0.90 (0.81, 1.00); 0.06	0.97 (0.86, 1.08); 0.55	0.50
**Reading (n = 9,618)**				
Far below grade (n = 272, 96, 176*)	20.6	28.1	16.5	
Somewhat below (n = 974, 370, 604)	16.8	21.9	13.7	
At grade level (n = 5,226, 2,452¸ 2,774)	17.8	21.0	14.9	
Somewhat above (n = 2,605, 1,469, 1,136)	18.2	21.2	14.2	
Far above grade (n = 541, 305¸ 236)	16.8	19.7	13.1	
**Basic Model** † Odds ratio (95% CI) per SD increase; P-value for trend	0.94 (0.88, 1.01); 0.11	0.93 (0.85, 1.02); 0.14	0.96 (0.87, 1.07); 0.49	0.71
**Adjusted Model** *^‡^* Odds ratio (95% CI) per SD increase; P-value for trend	0.93 (0.87, 1.00); 0.07	0.91 (0.83, 1.01); 0.09	0.95 (0.86, 1.06); 0.39	0.72
**Other subjects (n = 9,691)**				
Far below grade (n = 101, 28, 73*)	24.8	39.3	19.2	
Somewhat below (n = 476, 178, 298)	17.4	20.8	15.4	
At grade level (n = 5,921, 2,747, 3,174)	18.0	21.9	14.7	
Somewhat above (n = 2,887, 1,588, 1,299)	17.3	19.7	14.4	
Far above grade (n = 306, 172, 134)	14.7	19.2	9.0	
**Basic Model** † Odds ratio (95% CI) per SD increase; P-value for trend	0.87 (0.80, 0.95); 0.004	0.86 (0.77, 0.97); 0.02	0.89 (0.78, 1.01); 0.09	0.83
**Adjusted Model** *^‡^* Odds ratio (95% CI) per SD increase; P-value for trend	0.86 (0.79, 0.94); 0.003	0.86 (0.77, 0.97); 0.02	0.87 (0.76, 0.99); 0.05	0.98

† ORs adjusted for age, sex and cluster (polyclinic site). ^‡^ORs adjusted for age, sex, cluster (polyclinic site), treatment arm, child's BMI at age 6.5 years and number of older children in household * (n = x, y, z): x =  total number of children in group, y =  total number of females in group, z =  total number of males in group.

Academic performance measures have been categorized as “far below grade”, ”somewhat below”, “at grade level”, “somewhat above” and far above grade” for the presentation of results. In addition, academic performance was included as an ordered categorical variable in mixed-effects logistic regression models.

**Table 4** shows the results for the teacher- completed SDQ. In the fully-adjusted model in boys and girls combined, a SD increase in total difficulties score at 6.5 years was positively associated with ChEAT score ≥85^th^ percentile at 11.5 years (OR per SD increase in teacher-assessed total difficulties score  = 1.13; 1.07, 1.19). Associations were also observed between subcategories of the SDQ and ChEAT scores. The associations shown in **Table 4** were similar in boys and girls (p for sex-interaction ≥0.10). Similar results were found using the parent-assessed SDQ (**Table S2**).

**Table 4:** Association between Teacher Assessed Strengths and Difficulties Questionnnaire (SDQ) and ChEAT scores ≥85^th^ percentile.

Teacher SDQ scores §	Percentage of ChEAT scores ≥22.5			
	Basic Model†			
Emotional symptoms (n = 11,097)	Overall	Females	Males	P-value for sex*IQ interaction
Normal (0–4) (n = 9,885, 4,854, 5,031)*	17.9	21.4	14.6	
Borderline (5) (n = 569, 267, 302)	17.6	20.2	15.2	
Abnormal (6–10) (n = 643, 307, 336)	20.8	23.1	18.8	
**Basic Model** † Odds ratio (95% CI) per SD increase; P-value for trend	1.04 (0.99, 1.10); 0.13	1.00 (0.94, 1.08); 0.86	1.09 (1.01, 1.17); 0.05	0.10
**Adjusted Model** *^‡^* Odds ratio (95% CI) per SD increase; P-value for trend	1.06 (1.01, 1.12); 0.03	1.03 (0.96, 1.10); 0.44	1.10 (1.02, 1.20); 0.02	0.13
**Conduct problems (n = 11,098)**				
Normal (0–2) (n = 8,780, 4,741, 4,039)	18.0	20.9	14.7	
Borderline (3) (n = 994, 325, 669)	16.8	24.3	13.2	
Abnormal (4–10) (n = 1,324, 363, 961)	19.3	26.5	16.7	
**Basic Model** † Odds ratio (95% CI) per SD increase; P-value for trend	1.10 (1.04, 1.15); 0.002	1.12 (1.03, 1.21); 0.009	1.07 (1.00, 1.15); 0.07	0.46
**Adjusted Model** *^‡^* Odds ratio (95% CI) per SD increase; P-value for trend	1.09 (1.04, 1.15); 0.003	1.11 (1.03, 1.21); 0.01	1.07 (0.99, 1.15); 0.08	0.47
**Hyperactivity (n = 11,098)**				
Normal (0–5) (n = 8,208, 4,486, 3,722)	17.9	20.9	14.2	
Borderline (6) (n = 943, 366, 577)	18.8	23.2	15.9	
Abnormal (7–10) (n = 1,947, 577, 1370)	18.7	24.8	16.1	
**Basic Model** † Odds ratio (95% CI) per SD increase; P-value for trend	1.07 (1.02, 1.13); 0.01	1.07 (0.99, 1.15); 0.10	1.08 (1.00, 1.16); 0.07	0.79
**Adjusted Model** *^‡^* Odds ratio (95% CI) per SD increase; P-value for trend	1.08 (1.02, 1.13); 0.01	1.07 (0.99, 1.15); 0.10	1.08 (1.00, 1.17); 0.07	0.83
**Peer problems (n = 11,098)**				
Normal (0–3) (n = 8,561, 4,308, 4,253)	17.4	20.7	14.1	
Borderline (4) (n = 1,315, 599, 716)	18.2	21.4	15.5	
Abnormal (5–10) (n = 1,220, 521, 699)	22.8	27.8	19.0	
**Basic Model** † Odds ratio (95% CI) per SD increase; P-value for trend	1.13 (1.07, 1.19); <0.001	1.10 (1.03, 1.18); 0.01	1.15 (1.07, 1.25); 0.001	0.38
**Adjusted Model** *^‡^* Odds ratio (95% CI) per SD increase; P-value for trend	1.13 (1.07, 1.19); <0.001	1.10 (1.02, 1.18); 0.02	1.16 (1.07, 1.25); 0.001	0.33
**Total difficulties (n = 11,095)**				
Normal (0–11) (n = 7,462, 4,088, 3,374*)	17.5	20.5	14.0	
Borderline (12–15) (n = 1,937, 791, 1,146)	17.7	21.6	14.9	
Abnormal (16–40) (n = 1,696, 548, 1,148)	21.0	28.5	17.4	
**Basic Model** † Odds ratio (95% CI) per SD increase; P-value for trend	1.12 (1.06, 1.18); <0.001	1.10 (1.02, 1.18); 0.019	1.13 (1.05, 1.22); 0.003	0.49
**Adjusted Model** *^‡^* Odds ratio (95% CI) per SD increase; P-value for trend	1.13 (1.07, 1.19); <0.001	1.11 (1.03, 1.19); 0.008	1.14 (1.05, 1.23); 0.002	0.83

† ORs adjusted for age, sex and cluster (polyclinic site). ^‡^ORs adjusted for age, sex, cluster (polyclinic site), treatment arm, child's BMI at age 6.5 years and number of older children in household * (n = x, y, z): x =  total number of children in group, y =  total number of females in group, z =  total number of males in group.

§ Teacher SDQ associations also adjusted for teacher ID as a cluster variable.

Teacher SDQ measures have been categorized as “normal”, “borderline” and “abnormal”, according to standardized cut-off points for the SDQ, for the presentation of results, although SDQ score was included as a continuous, standardized variable in mixed-effects logistic regression models.

All associations remained when the more stringent criteria of a ChEAT score ≥91^st^ percentile was used as an indicator of problematic eating attitudes (**Tables S3–S6**), and when the polyclinic outlier was excluded (**Tables S7–S10**).

## Discussion

In our large, prospective cohort study, there was strong evidence that a higher IQ at age 6.5 years was inversely associated with problematic eating attitudes at 11.5 years among boys, but not girls. Academic performance in mathematics and “other subjects” (excluding mathematics, reading and writing) at age 6.5 years was inversely associated with problematic eating attitudes at age 11.5 years amongst both boys and girls, while behavioural difficulties at age 6.5 years were positively associated with problematic eating attitudes.

Our findings of a lack of association between IQ scales and problematic eating attitudes in girls are largely in accordance with some previous studies, which have found that individuals with eating disorders score either average or slightly above on general IQ tests, particularly among females. [13], [42] The finding that all forms of IQ (full-scale, verbal and performance) in early childhood were inversely associated with later problematic eating attitudes in boys appears to be novel. It may be that the school environment fosters a relationship between academic striving and body dissatisfaction and disordered eating among girls but not boys [43], counteracting an inverse association between IQ and ChEAT score. Alternatively, boys in higher IQ strata may be reluctant to acknowledge their problematic eating and so the results may be due to reporting bias by sex.[44] Otherwise, we may have detected a small, unimportant sex difference by chance, owing to our large sample size. This latter possibility is perhaps the most likely explanation, given that no evidence for a sex interaction was identified in the associations with academic performance, where an inverse relationship with problematic eating was found in both boys and girls.

In relation to our finding of an inverse relationship between academic performance in non-verbal subjects and problematic eating at age 11.5 years, non-verbal subjects draw more on abstract reasoning and visual-spatial abilities which may be impaired in individuals at risk of eating disorders, who frequently present with inflexible behaviours, rigid thinking patterns and impaired insight.[45] However, performance IQ, which similarly assesses these attributes, was not associated with risk of problematic eating in girls and there was also some weak evidence for an inverse association between ChEAT score and performance in verbal subjects.

In terms of the positive associations between behavioural difficulties and high ChEAT scores identified, behavioural problems and developmental delay have been associated with problematic eating in childhood, [46], [47] which may therefore act as a non-specific marker of underlying psychopathology [48]. In particular, negative emotions and low self-esteem are possible mechanisms by which high scores in SDQ domains (e.g. emotional symptoms and peer relationships) may increase the risk of future problematic eating attitudes. [11], [12], [17], [18]

Some of the dynamics of the relationships identified remain unresolved. For example, it is not known whether specific cognitive difficulties and behavioural problems, as measured here, independently increase risk of problematic eating or whether this relationship is confounded by other genetic or environmental risk factors that cause both eating and other cognitive or psychosocial problems.[10] Although our study controlled for a number of potential confounding factors associated with the outcome, associations are modest, and so it remains possible that residual confounding could explain the small changes in odds which are observed. Even without an assertion of causality, poor academic performance and behavioural difficulties at younger ages could be a manifestation of an underlying psychopathology related to problematic eating, and therefore early detection could lead to better preventative measures.

As we did not assess eating attitudes at age 6.5, the presence of pre-existing problematic eating attitudes at that age, which might have influenced both behavioural and cognitive development, cannot be ruled out.[49] It is also possible that associations of cognitive and behavioural measures with scores on ChEAT may reflect cognitive difficulties that influence an individual's ability to comprehend and accurately answer the ChEAT questionnaire. However, the ChEAT has exhibited internal and test-retest reliability among children as young as 8 years [31] which suggests that the test is likely to be valid.

A further issue relates to our timing and measurement of problematic eating attitudes. It is of note there is an inverse association between age and ChEAT in this sample, which is at odds with the fact that eating disorder prevalence typically increases with age over this developmental period. Similar trends have been found in a previous study [33] and these findings might suggest that younger children have more unhealthy eating attitudes than older children, or older children learn about eating disorders and realize that they shouldn't have “pathological” eating attitudes or at least do not admit to having them. Alternatively, these findings may be due to reduced validity of responses at younger ages. However, there is a narrow age range in our study (mean  = 11.6, SD  = 0.5, IQR  = 11.3–11.8) and the difference in the proportion of participants meeting the ChEAT 85^th^ cutoff between the age groups is quite small (1.8% between oldest and youngest tertiles).

Early adolescent eating attitudes do not necessarily lead to pathologic eating disorders in adolescence or adulthood. It is not entirely clear whether eating disorders occur along a spectrum of problematic eating attitudes or whether they are a separate entity.[50] The identification of child-onset eating disorders[51] as a separate category of eating disorder may mean risk factors influencing problematic eating in early adolescence are not the same as those which emerge in adolescence. However, problematic eating in early adolescence has been found to be associated with later eating disorders, even if it is not highly predictive.[4]–[7] In addition, we found similar trends when we used a higher ChEAT threshold score of ≥91^st^ percentile.

Given the criteria for entry into the study required full-term singleton infants weighing at least 2500g, the study sample is not representative of all live births in Belarus at the time of recruitment. However, trial staff estimate that only 1–2% of eligible women declined participation. Therefore, we have no reason to suspect any selection bias in estimates of the associations between ChEAT scores and other factors, though we acknowledge that the associations might vary in multiple, preterm or low-weight births which may limit the generalizability of our findings. Generalizability may also be limited by the loss to follow-up in this study, though any bias in the estimates obtained is likely to be minimal as differences between those included in the analyses compared to those with missing data were relatively small and the majority of the cohort were included in the analyses.[41]

The strengths of our study include its very large sample size, prospective design and excellent follow-up rate. In addition, rather than using specific neuropsychological tests to detect severe cognitive deficits, the use of general intelligence, academic and behavioural measures has enabled an assessment of subtle cognitive and behavioural difficulties which may influence risk of problematic eating in a general, healthy population. Our study is novel as it is the first one to examine prospective associations between both cognitive and behavioural difficulties with problematic eating in a middle-income country. It suggests that lower IQ, worse non-verbal academic performance and behavioural problems at early school age are positively associated with risk of problematic eating attitudes in early adolescence.

## Supporting Information

Table S1 **Association between ChEAT scores above 85^th^ percentile and potential confounders.**
(DOCX)

Table S2 **Association between Parent Strengths and Difficulties (SDQ) and ChEAT scores ≥85^th^ percentile.**
(DOCX)

Table S3 **Association between each IQ measure and ChEAT scores ≥91^st^ percentile.**
(DOCX)

Table S4 **Association between Teacher Assessed Academic Performance and ChEAT scores ≥91^st^ percentile.**
(DOCX)

Table S5 **Association between Teacher Assessed Strengths and Difficulties Questionnaire (SDQ) and ChEAT scores ≥91^st^ percentile.**
(DOCX)

Table S6 **Association between Parent Assessed Strengths and Difficulties Questionnaire (SDQ) and ChEAT scores ≥91st percentile.**
(DOCX)

Table S7 **Association between each IQ measure and ChEAT scores ≥85^th^ percentile, with exclusion of outlier polyclinic** ***^★^*** **.**
(DOCX)

Table S8 **Association between Teacher Assessed Academic Performance and ChEAT scores ≥85^th^ percentile, with exclusion of polyclinic outlier** ***^★^*** **.**
(DOCX)

Table S9 **Association between Teacher Assessed Academic Performance and ChEAT scores ≥85^th^ percentile, with exclusion of polyclinic outlier** ***^★^*** **.**
(DOCX)

Table S10 **Association between Parent Assessed Strengths and Difficulties (SDQ) and ChEAT scores ≥85^th^ percentile, with exclusion of polyclinic outlier** ***^★^*** **.**
(DOCX)
