# Supplementary material for: The Association of Early Childhood Cognitive Development and Behavioural Difficulties with Pre-Adolescent Problematic Eating Attitudes
Source: PLoS One. 2014 Aug 7;9(8):e104132. doi: 10.1371/journal.pone.0104132 (PMC4125275; doi:10.1371/journal.pone.0104132)
Supplement: Table S1 — Association between ChEAT scores above 85th percentile and potential confounders. (DOCX) [file pone.0104132.s001.docx]

**Table S1: Association between ChEAT scores above 85^th^ percentile and potential confounders**

| **Potential confounders** | **Percentage of ChEAT score ≥ 22.5** | **Odds Ratio, (95% CI)*^†^*; p-value** |
| --- | --- | --- |
| **Sex of child** |  |  |
| Females (n=6,675) | 20.8 | 1 (reference) |
| Males (n=7,076) | 14.1 | 0.62 (0.56, 0.68); <0.001 |
| **Age of child (years)** |  |  |
| 10.2-11.4 (n=4,582) | 18.4 |  |
| 11.4-11.7 (n =4,553) | 16.9 |  |
| 11.7-14.5 (n =4,555) | 16.6 | 0.90 (0.85, 0.96); 0.002 |
| **Treatment arm** |  |  |
| Control (n= 6,399) | 20.2 | 1 (reference) |
| Experimental (n=7,352) | 14.9 | 0.47 (0.23, 0.94); 0.04 |
| **Gestational age (weeks)** |  |  |
| 36-40 (n = 12,601) | 17.4 | 1 (reference) |
| 41-43 (n =1,150) | 16.6 | 1.04 (0.88, 1.24); 0.64 |
| **Birthweight (g)** |  |  |
| 2,500-3,250 (n =4,739) | 17.8 |  |
| 3,251-3,600 (n =4,611) | 17.4 |  |
| 3,602-5,650 (n = 4,401) | 16.8 | 1.00 (0.95, 1.06); 0.90 |
| **Apgar score at 5 minutes** |  |  |
| 1-8 (n= 5,768) | 17.5 | 1 (reference) |
| 9-10 (n= 7,983) | 17.2 | 1.04 (0.91, 1.17); 0.59 |
| **Mother’s age at birth (years)** |  |  |
| 14.2-22.1 (n = 4,586) | 17.8 |  |
| 22.1-26.4 (n = 4,583) | 17.4 |  |
| 26.4-47.1 (n= 4,582) | 16.8 | 0.96 (0.90, 1.01); 0.13 |
| **Maternal Education** |  |  |
| Incomplete Secondary or Common Secondary (n= 4,823) | 17.9 |  |
| Advanced Secondary or Partial University (n= 7,064) | 17.2 |  |
| Completed University (n= 1,864) | 16.2 | 1.00 (0.93, 1.07); 0.97 |
| **Paternal Education** |  |  |
| Incomplete Secondary or Common Secondary (n = 5,232) | 17.1 |  |
| Advanced Secondary or Partial University (n = 6,328) | 17.8 |  |
| Completed University (n = 1,756) | 16.2 | 1.02 (0.96, 1.09); 0.42 |
| **Highest household occupation** |  |  |
| Unemployed (n= 737) | 15.7 | 1 (reference) |
| Pupil/student (n = 178) | 19.1 | 1.43 (0.91, 2.23); 0.13 |
| Manual worker (n= 5571) | 17.9 | 1.08 (0.91, 2.24); 0.52 |
| Service worker (n= 7265) | 17.0 | 1.06 (0.85, 1.32); 0.59 |
| **Number of older children in household** |  |  |
| None (n= 7,827) | 18.1 |  |
| One (n= 4,779) | 16.3 |  |
| More than one (n= 1,145) | 16.4 | 0.90 (0.83, 0.97); 0.008 |
| **Smoking during pregnancy** |  |  |
| No (n= 13,466) | 17.3 | 1 (reference) |
| Yes (n= 285) | 19.0 | 0.93 (0.67, 1.27); 0.64 |
| **Urban vs rural** |  |  |
| Urban (n= 7,917) | 16.3 | 1 (reference) |
| Rural (n= 5,794) | 18.7 | 1.08 (0.51, 2.29); 0.84 |
| **Odds ratio** |  |  |
| **West vs East of Belarus** |  |  |
| West of Belarus (n = 7,200) | 16.1 | 1 (reference) |
| East of Belarus (n = 6,511) | 18.7 | 1.27 (0.60, 2.69); 0.53 |
| **Child’s BMI at PROBIT II (kg/m^2^)** |  |  |
| Lower tertile (n= 4,257) | 15.5 |  |
| Mid tertile (n= 4,252) | 15.6 |  |
| Upper tertile (n= 4,258) | 22.1 | 1.20 (1.17, 1.24); <0.001 |

*^†^ ORs adjusted for sex, age and clustering by hospital/polyclinic. For categorical variables, ORs are per level increase in the category*
