# Supplementary material for: The Association of Early Childhood Cognitive Development and Behavioural Difficulties with Pre-Adolescent Problematic Eating Attitudes
Source: PLoS One. 2014 Aug 7;9(8):e104132. doi: 10.1371/journal.pone.0104132 (PMC4125275; doi:10.1371/journal.pone.0104132)
Supplement: Table S2 — Association between Parent Strengths and Difficulties (SDQ) and ChEAT scores ≥85th percentile. (DOCX) [file pone.0104132.s002.docx]

**Table S2: Association between Parent Strengths and Difficulties (SDQ) and ChEAT scores ≥85^th^ percentile**

| **Parent SDQ scores** | **Percentage of ChEAT scores ≥ 22.5** | | | |
| --- | --- | --- | --- | --- |
| **Conduct problems (n=12,654)** | **Overall** | **Females** | **Males** | **P-value for sex*IQ interaction** |
| Normal (0-2) (n=9,593, 4,938, 4,655) | 17.5 | 20.4 | 14.5 |  |
| Borderline (3) (n=1,677, 708, 969) | 16.5 | 22.0 | 12.4 |  |
| Abnormal (4-10) (n=1,384, 520, 864) | 20.2 | 27.7 | 15.7 |  |
| **Basic Model** *^†^* Odds ratio (95% CI) per SD increase; P-value for trend | 1.04 (0.99, 1.09); 0.10 | 1.08 (1.02, 1.16); 0.02 | 1.00 (0.93, 1.07); 0.97 | 0.10 |
| **Adjusted Model** *^‡^* Odds ratio (95% CI) per SD increase; P-value for trend | 1.04 (0.99, 1.09); 0.14 | 1.08 (1.01, 1.15); 0.04 | 1.00 (0.93, 1.07); 0.96 | 0.13 |
| **Hyperactivity (n=12,655)** |  |  |  |  |
| Normal (0-5) (n=8,104, 4,285, 3,819) | 17.9 | 21.1 | 14.3 |  |
| Borderline (6) (n=1,828, 782, 1,046) | 16.6 | 19.8 | 14.2 |  |
| Abnormal (7-10) (n=2,723, 1,099, 1,624) | 17.7 | 22.3 | 14.5 |  |
| **Basic Model** *^†^* Odds ratio (95% CI) per SD increase; P-value for trend | 1.05 (1.00, 1.11); 0.04 | 1.06 (0.99, 1.13); 0.09 | 1.05 (0.98, 1.13); 0.18 | 0.90 |
| **Adjusted Model** *^‡^* Odds ratio (95% CI) per SD increase; P-value for trend | 1.05 (1.00, 1.10); 0.07 | 1.05 (0.99, 1.13); 0.11 | 1.04 (0.97, 1.12); 0.29 | 0.80 |
| **Peer problems (n=12,656)** |  |  |  |  |
| Normal (0-2) (n=6,634, 3,247, 3,387*) | 16.4 | 19.9 | 13.1 |  |
| Borderline (3) (n=2,595, 1,299, 1,296) | 19.0 | 22.4 | 15.5 |  |
| Abnormal (4-10) (n=3,427, 1,621, 1,806) | 19.1 | 22.9 | 15.8 |  |
| **Basic Model** *^†^* Odds ratio (95% CI) per SD increase; P-value for trend | 1.08 (1.03, 1.14); 0.003 | 1.08 (1.01, 1.16); 0.03 | 1.09 (1.01, 1.17); 0.03 | 0.84 |
| **Adjusted Model** *^‡^* Odds ratio (95% CI) per SD increase; P-value for trend | 1.08 (1.03, 1.13); 0.005 | 1.07 (1.00, 1.14); 0.06 | 1.09 (1.01, 1.17); 0.03 | 0.70 |
| **Total difficulties (n= 12,651)** |  |  |  |  |
| Normal (0-13) (n=8,519, 4,309, 4,210) | 16.9 | 20.0 | 13.6 |  |
| Borderline (14-16) (n=2,140, 1,013, 1,127) | 19.1 | 22.6 | 15.9 |  |
| Abnormal (17-40) (n=1,992, 841, 1,151) | 19.6 | 25.3 | 15.5 |  |
| **Basic Model** *^†^* Odds ratio (95% CI) per SD increase; P-value for trend | 1.09 (1.04, 1.15); 0.001 | 1.10 (1.04, 1.18); 0.005 | 1.08 (1.00, 1.16); 0.04 | 0.76 |
| **Adjusted Model** *^‡^* Odds ratio (95% CI) per SD increase; P-value for trend | 1.09 (1.04, 1.14); 0.002 | 1.10 (1.03, 1.18); 0.007 | 1.08 (1.00, 1.16); 0.05 | 0.77 |

*^†^ ORs adjusted for age, sex and cluster (polyclinic site).* *^‡^ ORs adjusted for age, sex, cluster (polyclinic site), treatment arm, child’s BMI at age 6.5 years and number of older children in household. * (n=x, y, z): x= total number of children in group, y= total number of females in group, z= total number of males in group.*

*Parent SDQ measures have been categorized as “normal”, “borderline” and “abnormal”, according to standardized cut-off points for the SDQ, for the presentation of results, although SDQ score was included as a continuous, standardized variable in mixed-effects logistic regression models.*
