# Supplementary material for: The Association of Early Childhood Cognitive Development and Behavioural Difficulties with Pre-Adolescent Problematic Eating Attitudes
Source: PLoS One. 2014 Aug 7;9(8):e104132. doi: 10.1371/journal.pone.0104132 (PMC4125275; doi:10.1371/journal.pone.0104132)
Supplement: Table S3 — Association between each IQ measure and ChEAT scores ≥91st percentile. (DOCX) [file pone.0104132.s003.docx]

**Table S3: Association between each IQ measure and ChEAT scores ≥91^st^ percentile**

| **IQ Measures** | **Percentage of ChEAT scores ≥ 25.5** | | | |
| --- | --- | --- | --- | --- |
| **Full IQ (n=12,663)** | **Overall** | **Females** | **Males** | **P-value for sex*IQ interaction** |
| Below average (n=2,083, 941, 1,142*) | 11.6 | 15.3 | 8.6 |  |
| Average (n=6,019, 3,049, 2,970) | 10.8 | 13.2 | 8.4 |  |
| Above average (n=4,561, 2,176, 2,385) | 10.9 | 14.0 | 8.1 |  |
| **Basic Model** *^†^* Odds ratio (95% CI) per SD increase; P-value for trend | 0.95 (0.88, 1.02); 0.17 | 0.98 (0.90, 1.08); 0.73 | 0.91 (0.82, 1.01); 0.10 | 0.44 |
| **Adjusted Model** *^‡^* Odds ratio (95% CI) per SD increase; P-value for trend | 0.93 (0.86, 0.99); 0.040 | 0.96 (0.87,1.05); 0.39 | 0.89 (0.80, 0.99); 0.039 | 0.45 |
| **Verbal IQ (n=12,667)** |  |  |  |  |
| Below average (n=2,783, 1,258, 1,525) | 11.1 | 14.6 | 8.3 |  |
| Average (n=5,752, 2,915, 2,837) | 10.7 | 13.2 | 8.2 |  |
| Above average (n=4,132, 1,994, 2,138) | 11.3 | 14.2 | 8.5 |  |
| **Basic Model** *^†^* Odds ratio (95% CI) per SD increase; P-value for trend | 0.97 (0.90, 1.03); 0.34 | 0.97 (0.89, 1.07); 0.63 | 0.95 (0.86, 1.06); 0.38 | 0.74 |
| **Adjusted Model** *^‡^* Odds ratio (95% CI) per SD increase; P-value for trend | 0.93 (0.87, 1.00); 0.07 | 0.95 (0.87, 1.04); 0.28 | 0.92 (0.83, 1.03); 0.15 | 0.69 |
| **Performance IQ (n=12,675)** |  |  |  |  |
| Below average (n=1,314, 583, 731) | 12.3 | 17.2 | 8.3 |  |
| Average (n=7,145, 3,617, 3,528) | 10.7 | 12.8 | 8.5 |  |
| Above average (n=4,216, 1,971, 2,245) | 11.1 | 14.6 | 8.1 |  |
| **Basic Model** *^†^* Odds ratio (95% CI) per SD increase; P-value for trend | 0.95 (0.89, 1.01); 0.12 | 1.00 (0.91, 1.09); 0.96 | 0.89 (0.80, 0.98); 0.027 | 0.02 |
| **Adjusted Model** *^‡^* Odds ratio (95% CI) per SD increase; P-value for trend | 0.94 (0.88, 1.00); 0.07 | 0.98 (0.90, 1.08); 0.74 | 0.88 (0.80, 0.98); 0.022 | 0.02 |

*^†^ ORs adjusted for age, sex and cluster (polyclinic site).* *^‡^ ORs adjusted for age, sex, cluster (polyclinic site), treatment arm, child’s BMI at age 6.5 years and number of older children in household. * (n=x, y, z): x= total number of children in group, y= total number of females in group, z= total number of males in group.*

*IQ measures have been categorized as “below average” (<90), “average” (90-109) and “above average”(>109), according to Weschler scale IQ classifications, for the presentation of results, although IQ was included as a continuous, standardized variable in mixed-effects logistic regression models.*
