# Supplementary material for: The Association of Early Childhood Cognitive Development and Behavioural Difficulties with Pre-Adolescent Problematic Eating Attitudes
Source: PLoS One. 2014 Aug 7;9(8):e104132. doi: 10.1371/journal.pone.0104132 (PMC4125275; doi:10.1371/journal.pone.0104132)
Supplement: Table S4 — Association between Teacher Assessed Academic Performance and ChEAT scores ≥91st percentile. (DOCX) [file pone.0104132.s004.docx]

**Table S4: Association between Teacher Assessed Academic Performance and ChEAT scores ≥91^st^ percentile**

| **Academic subject** | **Percentage of ChEAT scores ≥ 25.5** | | | | | **Odds ratio (95% CI) per category increase;**  **P-value for trend** | |
| --- | --- | --- | --- | --- | --- | --- | --- |
|  | **Far below grade** | **Somewhat below** | **At grade level** | **Somewhat above** | **Far above grade** | **Basic model***^†^* | **Adjusted model***^‡^* |
| Mathematics (n=9,954) | 14.5 (n=248*) | 10.6 (n=1,055) | 11.7 (n=5,369) | 10.4 (n=2,873) | 9.1 (n=409) | 0.89 (0.81, 0.97); 0.010 | 0.88 (0.80, 0.96); 0.006 |
| Writing (n=9,760) | 10.4 (n=269) | 10.8 (n=1,009) | 11.1 (n=5,707) | 11.7 (n=2,433) | 9.7 (n=342) | 0.94 (0.86, 1.03); 0.19 | 0.93 (0.85, 1.02); 0.12 |
| Reading (n=9,618) | 12.9 (n=272) | 10.3 (n=974) | 11.0 (n=5,226) | 11.8 (n=2,605) | 9.8 (n=541) | 0.94 (0.87, 1.02); 0.17 | 0.93 (0.86, 1.02); 0.13 |
| Other subjects (n=9,691) | 14.9 (n=101) | 10.9 (n=476) | 11.4 (n=5,921) | 10.8 (n=2,887) | 8.8 (n=306) | 0.86 (0.78, 0.96); 0.009 | 0.85 (0.77, 0.95); 0.007 |

*^†^ ORs adjusted for age, sex and cluster (polyclinic site).* *^‡^ ORs adjusted for age, sex, cluster (polyclinic site), treatment arm, child’s BMI at age 6.5 years and number of older children in household. *(n=x): x= total number of children in group. Results are not stratified by sex as there was no evidence for a sex interaction in the association between academic performance and ChEAT score in the main analysis*

*Academic performance measures have been categorized as “far below grade”, ”somewhat below”, “at grade level”, “somewhat above” and far above grade” for the presentation of results. In addition, academic performance was included as an ordered categorical variable in mixed-effects logistic regression models.*
