# Supplementary material for: The Association of Early Childhood Cognitive Development and Behavioural Difficulties with Pre-Adolescent Problematic Eating Attitudes
Source: PLoS One. 2014 Aug 7;9(8):e104132. doi: 10.1371/journal.pone.0104132 (PMC4125275; doi:10.1371/journal.pone.0104132)
Supplement: Table S5 — Association between Teacher Assessed Strengths and Difficulties Questionnaire (SDQ) and ChEAT scores ≥91st percentile. (DOCX) [file pone.0104132.s005.docx]

**Table S5: Association between Teacher Assessed Strengths and Difficulties Questionnaire (SDQ) and ChEAT scores ≥91^st^ percentile**

| **Teacher SDQ scores^§^** | **Percentage of ChEAT scores ≥ 25.5** | | | **Odds ratio (95% CI) per SD increase; P-value for trend** | |
| --- | --- | --- | --- | --- | --- |
|  | **Normal** | **Borderline** | **Abnormal** | **Basic model***^†^* | **Adjusted model***^‡^* |
| Emotional Symptoms (N=11,097) | 11.1 (n=9,885*) | 11.3 (n=569) | 12.9 (n=643) | 1.06 (1.00, 1.13); 0.07 | 1.07 (1.02, 1.15); 0.02 |
| Conduct Problems (N=11,098) | 11.3 (n=8,780) | 9.5 (n=994) | 12.1 (n=1,324) | 1.11(1.04, 1.18); 0.003 | 1.11 (1.04, 1.18); 0.003 |
| Hyperactivity (N=11,098) | 10.9 (n=8,208) | 12.2 (n=943) | 12.1 (n=1,947) | 1.09 (1.02, 1.16); 0.01 | 1.09 (1.03, 1.17); 0.01 |
| Peer problems (N=11,096) | 10.8 (n=8,561) | 12.2 (n=1,315) | 13.4 (n=1,220) | 1.13 (1.07, 1.21); <0.001 | 1.13 (1.06, 1.20); <0.001 |
| Total difficulties score (N=11,095) | 10.8 (n=7,462) | 11.3 (n=1,937) | 13.1 (n=1,696) | 1.14 (1.07, 1.21); <0.001 | 1.15 (1.08, 1.22); <0.001 |

*^†^ ORs adjusted for age, sex and cluster (polyclinic site).* *^‡^ ORs adjusted for age, sex, cluster (polyclinic site), treatment arm, child’s BMI at age 6.5 years and number of older children in household. *(n=x): x= total number of children in group.* **^§^** *Teacher SDQ associations also adjusted for teacher ID as a cluster variable.* *Results are not stratified by sex as there was no evidence for a sex interaction in the association between teacher SDQ score and ChEAT score in the main analysis.*

*Teacher SDQ measures have been categorized as “normal”, “borderline” and “abnormal”, according to standardized cut-off points for the SDQ, for the presentation of results, although SDQ score was included as a continuous, standardized variable in mixed-effects logistic regression models.*
