# Supplementary material for: The Association of Early Childhood Cognitive Development and Behavioural Difficulties with Pre-Adolescent Problematic Eating Attitudes
Source: PLoS One. 2014 Aug 7;9(8):e104132. doi: 10.1371/journal.pone.0104132 (PMC4125275; doi:10.1371/journal.pone.0104132)
Supplement: Table S6 — Association between Parent Assessed Strengths and Difficulties Questionnaire (SDQ) and ChEAT scores ≥91st percentile. (DOCX) [file pone.0104132.s006.docx]

**Table S6: Association between Parent Assessed Strengths and Difficulties Questionnaire (SDQ) and ChEAT scores ≥91st percentile**

| **Parent SDQ scores** | **Percentage of ChEAT scores ≥ 25.5** | | | **Odds ratio (95% CI) per SD increase; P-value for trend** | |
| --- | --- | --- | --- | --- | --- |
|  | **Normal** | **Borderline** | **Abnormal** | **Basic model***^†^* | **Adjusted model***^‡^* |
| Emotional Symptoms (N=12,652) | 10.6 (n=8,843*) | 11.3 (n=1,593) | 12.6 (n=2,216) | 1.08 (1.02, 1.14); 0.01 | 1.09 (1.03, 1.15); 0.006 |
| Conduct Problems (N=12,654) | 10.9 (n=9,593) | 10.3 (n=1,677) | 12.8 (n=1,384) | 1.04 (0.98, 1.10); 0.19 | 1.04 (0.98, 1.10); 0.25 |
| Hyperactivity (N=12,655) | 11.3 (n=8,104) | 10.5 (n=1,828) | 10.6 (n= 2,723) | 1.04 (0.98, 1.10); 0.25 | 1.03 (0.97, 1.09); 0.33 |
| Peer problems (N=12,656) | 10.1 (n=6,634) | 11.7 (n=2,595) | 12.2 (n=3,427) | 1.10 (1.04, 1.17); 0.002 | 1.10 (1.04, 1.16); 0.004 |
| Total difficulties* (N=12,651) | 10.5 (n=8,519) | 11.4 (n=2,140) | 12.6 (n=1,992) | 1.10 (1.04, 1.16); 0.004 | 1.09 (1.03, 1.16); 0.005 |

*^†^ ORs adjusted for age, sex and cluster (polyclinic site).* *^‡^ ORs adjusted for age, sex, cluster (polyclinic site), treatment arm, child’s BMI at age 6.5 years and number of older children in household. *(n=x): x= total number of children in group. Results are not stratified by sex as there was no evidence for a sex interaction in the association between parent SDQ score and ChEAT score in the main analysis*

*Parent SDQ measures have been categorized as “normal”, “borderline” and “abnormal”, according to standardized cut-off points for the SDQ, for the presentation of results, although SDQ score was included as a continuous, standardized variable in mixed-effects logistic regression models.*
