# Supplementary material for: The Association of Early Childhood Cognitive Development and Behavioural Difficulties with Pre-Adolescent Problematic Eating Attitudes
Source: PLoS One. 2014 Aug 7;9(8):e104132. doi: 10.1371/journal.pone.0104132 (PMC4125275; doi:10.1371/journal.pone.0104132)
Supplement: Table S7 — Association between each IQ measure and ChEAT scores ≥85th percentile, with exclusion of outlier polyclinic ★ . (DOCX) [file pone.0104132.s007.docx]

**Table S7: Association between each IQ measure and ChEAT scores ≥85^th^ percentile, with exclusion of outlier polyclinic** *^★^*

| **IQ Measures** | **Percentage of ChEAT scores ≥ 22.5** | | | |
| --- | --- | --- | --- | --- |
|  | **Basic Model***^†^* | | | |
| **Full IQ (n=12,111)** | **Overall** | **Females** | **Males** | **P-value for sex*IQ interaction** |
| Below average (n=1,973, 896, 1,077*) | 19.3 | 23.2 | 16.1 |  |
| Average (n=5,743, 2,920, 2,823) | 18.1 | 21.0 | 15.2 |  |
| Above average (n=4,395, 2,101, 2,294) | 18.1 | 22.6 | 14.1 |  |
| **Basic Model** *^†^*Odds ratio (95% CI) per SD increase; P-value for trend | 0.97 (0.92, 1.03); 0.37 | 1.05 (0.97, 1.14); 0.24 | 0.89 (0.82, 0.97); 0.01 | 0.01 |
| **Adjusted Model** *^‡^* Odds ratio (95% CI) per SD increase; P-value for trend | 0.95 (0.89, 1.00); 0.07 | 1.02 (0.94, 1.11); 0.61 | 0.86 (0.80, 0.95); 0.002 | 0.01 |
| **Verbal IQ (n=12,114)** |  |  |  |  |
| Below average (n=2,652, 1,203, 1,449*) | 19.3 | 22.6 | 16.5 |  |
| Average (n=5,508, 2,803, 2,705) | 17.8 | 20.7 | 14.8 |  |
| Above average (n=3,954, 1,912, 2,042) | 18.5 | 23.2 | 14.1 |  |
| **Basic Model** *^†^* Odds ratio (95% CI) per SD increase; P-value for trend | 0.98 (0.93, 1.04); 0.60 | 1.05 (0.97, 1.13); 0.27 | 0.92 (0.84, 1.00); 0.06 | 0.04 |
| **Adjusted Model** *^‡^* Odds ratio (95% CI) per SD increase; P-value for trend | 0.95 (0.90, 1.01); 0.10 | 1.01 (0.94, 1.10); 0.73 | 0.88 (0.81, 0.96); 0.01 | 0.03 |
| **Performance IQ (n= 12,123)** |  |  |  |  |
| Below average (n=1,218, 549, 669*) | 20.9 | 26.8 | 16.0 |  |
| Average (n=6,835, 3,472, 3,363) | 18.1 | 20.6 | 15.5 |  |
| Above average (n=4,070, 1,901, 2,169) | 18.0 | 22.8 | 13.7 |  |
| **Basic Model** *^†^* Odds ratio (95% CI) per SD increase; P-value for trend | 0.97 (0.92, 1.02); 0.25 | 1.04 (0.96, 1.12); 0.33 | 0.89 (0.82, 0.96); 0.008 | 0.003 |
| **Adjusted Model** *^‡^* Odds ratio (95% CI) per SD increase; P-value for trend | 0.96 (0.90, 1.01); 0.13 | 1.02 (0.95, 1.11); 0.55 | 0.88 (0.81, 0.96); 0.006 | 0.004 |

*^†^ ORs adjusted for age, sex and cluster (polyclinic site).* *^‡^ ORs adjusted for age, sex, cluster (polyclinic site), treatment arm, child’s BMI at age 6.5 years and number of older children in household. * (n=x, y, z): x= total number of children in group, y= total number of females in group, z= total number of males in group. ^★^Intervention site where 75% of respondents answered “never” to all 24 items of the ChEAT questionnaire*

*IQ measures have been categorized as “below average” (<90), “average” (90-109) and “above average”(>109), according to Weschler scale IQ classifications, for the presentation of results, although IQ was included as a continuous, standardized variable in mixed-effects logistic regression models.*
