# Supplementary material for: The Association of Early Childhood Cognitive Development and Behavioural Difficulties with Pre-Adolescent Problematic Eating Attitudes
Source: PLoS One. 2014 Aug 7;9(8):e104132. doi: 10.1371/journal.pone.0104132 (PMC4125275; doi:10.1371/journal.pone.0104132)
Supplement: Table S9 — Association between Teacher Assessed Academic Performance and ChEAT scores ≥85th percentile, with exclusion of polyclinic outlier ★ . (DOCX) [file pone.0104132.s009.docx]

**Table S9: Association between Teacher Assessed Academic Performance and ChEAT scores ≥85^th^ percentile, with exclusion of polyclinic outlier***^★^*

| **Teacher SDQ scores^§^** | **Percentage of ChEAT scores ≥ 22.5** | | | **Odds ratio (95% CI) per SD increase; P-value for trend** | |
| --- | --- | --- | --- | --- | --- |
|  | **Normal** | **Borderline** | **Abnormal** | **Basic model***^†^* | **Adjusted model***^‡^* |
| Emotional Symptoms (N=10,723) | 18.5 (n=9,563*) | 18.2 (n=543) | 21.6 (n=617) | 1.04 (0.99, 1.10); 0.14 | 1.06 (1.01, 1.12); 0.03 |
| Conduct Problem (N=10,723) | 18.6 (n=8,488) | 17.3 (n=958) | 20.0 (n=1,277) | 1.09 (1.04, 1.15); 0.002 | 1.09 (1.03, 1.15); 0.003 |
| Hyperactivity (N=10,723) | 18.5 (n=7,931) | 19.4 (n=909) | 19.2 (n=1,883) | 1.07 (1.02, 1.13); 0.02 | 1.07 (1.02, 1.13); 0.02 |
| Peer problems (N=10,721) | 18.0 (n=8,267) | 18.9 (n=1,266) | 23.3 (n=1,188) | 1.13 (1.07, 1.19); <0.001 | 1.13 (1.07, 1.19); <0.001 |
| Total difficulties^#^ (N=10,721) | 18.1 (n=7,204) | 18.2 (n=1,879) | 21.6 (n=1,638) | 1.12 (1.06, 1.18); <0.001 | 1.12 (1.07, 1.19); <0.001 |

*^†^ ORs adjusted for age, sex and cluster (polyclinic site).* *^‡^ ORs adjusted for age, sex, cluster (polyclinic site), treatment arm, child’s BMI at age 6.5 years and number of older children in household. *(n=x): x= total number of children in group. ^★^Intervention site where 75% of respondents answered “never” to all 24 items of the ChEAT questionnaire*

**^§^** *Teacher SDQ associations also adjusted for teacher ID as a cluster variable.* *Results are not stratified by sex as there was no evidence for a sex interaction in the association between teacher SDQ score and ChEAT score in the main analysis.*

*Teacher SDQ measures have been categorized as “normal”, “borderline” and “abnormal”, according to standardized cut-off points for the SDQ, for the presentation of results, although SDQ score was included as a continuous, standardized variable in mixed-effects logistic regression models.*
