# Supplementary material for: The Association of Early Childhood Cognitive Development and Behavioural Difficulties with Pre-Adolescent Problematic Eating Attitudes
Source: PLoS One. 2014 Aug 7;9(8):e104132. doi: 10.1371/journal.pone.0104132 (PMC4125275; doi:10.1371/journal.pone.0104132)
Supplement: Table S10 — Association between Parent Assessed Strengths and Difficulties (SDQ) and ChEAT scores ≥85th percentile, with exclusion of polyclinic outlier ★ . (DOCX) [file pone.0104132.s010.docx]

**Table S10: Association between Parent Assessed Strengths and Difficulties (SDQ) and ChEAT scores ≥85^th^ percentile, with exclusion of polyclinic outlier***^★^*

| **Parent SDQ scores** | **Percentage of ChEAT scores ≥ 22.5** | | | **Odds ratio (95% CI) per SD increase; P-value for trend** | |
| --- | --- | --- | --- | --- | --- |
|  | **Normal** | **Borderline** | **Abnormal** | **Basic model***^†^* | **Adjusted model***^‡^* |
| Emotional Symptoms (N=12,101) | 17.6 (n=8,477*) | 20.2 (n=1,508) | 19.9 (n=2,116) | 1.06 (1.01, 1.12); 0.02 | 1.07 (1.02, 1.12); 0.008 |
| Conduct Problems (N=12,103) | 18.2 (n=9,177) | 17.2 (n=1,602) | 21.0 (n=1,324) | 1.05 (1.00, 1.10); 0.07 | 1.04 (0.99, 1.09); 0.10 |
| Hyperactivity (N=12,103) | 18.6 (n=7,775) | 17.4 (n=1,720) | 18.4 (n=2,608) | 1.05 (1.00, 1.11); 0.05 | 1.05 (1.00, 1.10); 0.08 |
| Peer problems (N=12,104) | 17.1 (n=6,304) | 19.5 (n=2,505) | 19.9 (n=3,295) | 1.09 (1.04, 1.14); 0.002 | 1.08 (1.03, 1.14); 0.003 |
| Total difficulties^#^ (N=12,100) | 17.5 (n=8,159) | 19.9 (n=2,043) | 20.4 (n=1,898) | 1.09 (1.04, 1.15); 0.001 | 1.09 (1.04, 1.15); 0.001 |

*^†^ ORs adjusted for age, sex and cluster (polyclinic site).* *^‡^ ORs adjusted for age, sex, cluster (polyclinic site), treatment arm, child’s BMI at age 6.5 years and number of older children in household. *(n=x): x= total number of children in group. ^★^Intervention site where 75% of respondents answered “never” to all 24 items of the ChEAT questionnaire*

*N.B. results are not stratified by sex as there was no evidence for a sex interaction in the association between parent SDQ score and ChEAT score in the main analysis*

*Parent-assessed SDQ measures have been categorized as “normal”, “borderline” and “abnormal”, according to standardized cut-off points for the SDQ, for the presentation of results, although SDQ score was included as a continuous, standardized variable in mixed-effects logistic regression models.*
